# Ventricular Septal Defect: the Three-Dimensional Point of View

**Published:** 2013-05-06

**Authors:** V Parisi, E Ratto, C Silvestri, F Pastore

**Affiliations:** 1 Department of Clinical Medicine, Cardiovascular and Immunological Sciences, University of Naples “Federico II”; 2 Department of Cardiology, AOU “Maggiore Della Carità”, Novara

**Keywords:** Three Dimensional Transthoracic Echocardiography, Aortic Regurgitation, Congenital Heart Defects

## Abstract

This case highlights the clinical usefulness of three-dimensional (3D) echocardiography. The diagnosis of inter-ventricular septal defect associated with aortic regurgitation has been performed in a 50-year-old man using 3D echocardiography. This advanced echocardiography could accurately reproduce the anatomy of the defect and provide further insights in the mechanisms of aortic regurgitation showing an unusual non-coronary cusp prolapse. The routinely use of 3D echocardiography in clinics might allow a better characterization of cardiac anatomy, especially of aortic valve disorders.

## CASE REPORT

In a 50-year-old man, an echocardiographic study has been performed because of the clinical evidence of a 3/6 systolic-diastolic murmur. He has no cardiovascular history with class NYHA I.

Two-dimensional (2D) echocardiography has noticed a restrictive inter-ventricular septal defect associated with moderate aortic regurgitation, with evidence of a calcification of the non-coronary cusp.

**Figure 1.**
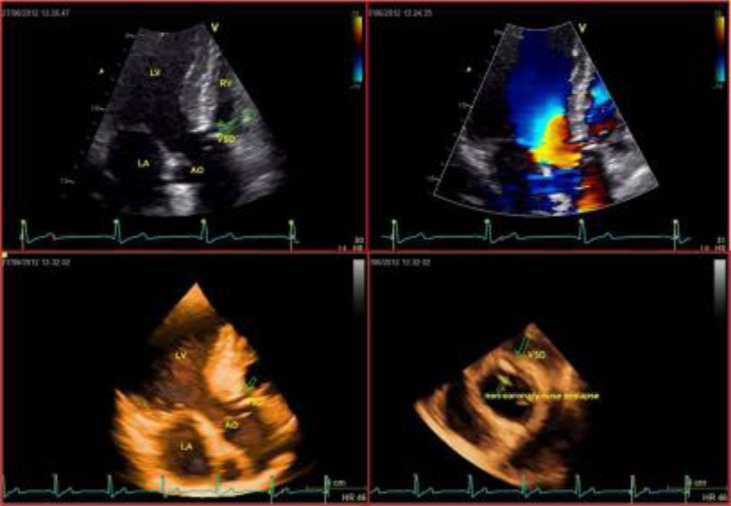
**
Echocardiographic Images** .LV: left ventricle; RV: right ventricle; LA: left atrium; AO: aorta: VSD: ventricular septal defect. A) 2D apical long axis view showing the vertricular septal defect. B) 2D color apical long axis view demonstrating the interventricular shunt. C) 3D apical long axis view, perfectly reproducing the septal defect. D) 3D short axis view showing the ventricular septal defect, the degeneration and prolapse of the non-coronary aortic cusp.

The mechanism of aortic regurgitation could not be elucidated. Three-dimensional (3D) trans-thoracic echocardiography can reproduce the anatomy of the defect and give further insights in the mechanisms of aortic regurgitation showing a non-coronary cusp prolapse. The image ( Figure 1 ) shows the exact correspondence between 2D (A) and 3D (C) echocardiography in the identification of the ventricular septal defect. The 3D image has an incredible anatomical accuracy. The color-echocardiogram (B) shows the inter-ventricular shunts and the 3D short axis (D) confirms the presence of the defect and shows the non-coronary cusp degeneration and prolapse. Three-dimensional echocardiography is already the echocardiographic modality of choice for establishing the diagnosis and guide therapy in mitral valve diseases [ 1 , 2 ] and allows detailed anatomical and functional assessment of various cardiac disorders. In this case, 3D echocardiography is particularly useful in the identification of the anatomical and functional characteristics of a ventricular septal defect with associated aortic regurgitation unusually related to non coronary-cups prolapse; in fact, right coronary cusp prolapse is usually more common [ 3 ] .

In a recent review Charakida et al has showed the power of 3D echocardiography in the characterization of the endovascular closure of the inter-ventricular septal defect. 3D Echo allows the visualization of the ventricular septum in any desired orientation. Using these images, the operator can choose more suitable devices and reduce the risk of complications, such as aortic and tricuspid damages [ 4 ] .

In these years, there have been many innovations in echocardiographic imaging. The use of the Speckle Strain consents to assess tissue deformation and the contrast-echocardiography gives more detailed images. According to us, 3D echocardiography is the most helpful echocardiographic change because it improves the quality of imaging replacing more invasive or expensive techniques such as trans-esophageal Echocardiography and Magnetic Resonance.

This method is cheap, repeatable even if it is highly depending on the ability of the operator, hence the learning curve is particularly crucial. In spite of this, it has to be considered the future echocardiographic method, especially where it is necessary to consider the relationship between costs and benefits, as in the smallest hospital.

3DE measures chamber size, ventricular mass and function with the same accuracy and reproducibility as CMR and radionuclide ventriculography. The accuracy for ventricular function is essential when such measurements regulate the use of an expensive device therapy (cardiac resynchronization and implantable cardio-defibrillator therapy).

This case report underlines the 3D echocardiography importance for diagnose of inter-ventricular septal defect. We want to highlight the future importance of the safe, cheap, repeatable, accurate and non-invasive diagnostic methods.
